# Lecanemab Slows Amyloid‐Induced Tau Pathology as Supported by CSF MTBR‐tau243 in Clarity AD

**DOI:** 10.1002/alz.095507

**Published:** 2025-01-09

**Authors:** Kristin R Wildsmith, Pallavi Sachdev, Kanta Horie, Larisa Reyderman, Arnaud Charil, Michio Kanekiyo, Han Yin, David Li, Akihiko Koyama, Shobha Dhadda, Michael C. Irizarry, Lynn D Kramer

**Affiliations:** ^1^ Eisai Inc., Nutley, NJ USA; ^2^ Eisai Co, Tsukuba Japan

## Abstract

**Background:**

Lecanemab is a humanized immunoglobulin G1 monoclonal antibody targeting neurotoxic Aβ protofibrils and Aβ plaques, which reduces markers of amyloid and significantly slows clinical decline on multiple cognition and function measures. Cerebrospinal fluid (CSF) microtubule‐binding region (MTBR) of tau containing the residue 243 (MTBR‐tau243) is an emerging tau pathology biomarker that tracks with tangle formation in Alzheimer’s disease (AD) [Horie et al. Nat Med. 2023;29:1954‐1963]. In order to evaluate effects of lecanemab on MTBR‐tau243, CSF was analyzed in a subset of early AD participants from the phase 3 (Clarity AD) trial.

**Method:**

CSF from n = 167 early AD participants from Clarity AD was analyzed using an IP‐MS based method [Horie et al. Nat Med. 2023;29:1954‐1963.]. Pearson correlation analysis was performed to assess relationships between CSF MTBR‐tau243, AD fluid biomarkers, amyloid PET and tau PET SUVR in multiple regions of interest. Mixed model for repeated measures was used to analyze change from baseline and assess treatment effect over 18 months.

**Result:**

Baseline correlation analysis show that CSF MTBR‐tau243 has the highest correlation with Tau PET SUVR across regions of interest (ROI) (Braak 1‐6, whole cortical grey (WCG) vs. all other fluid biomarkers assessed (CSF markers: ptau181, total tau, neurogranin, NfL, Aβ40 and Aβ42; plasma markers: pTau217, pTau181, Aβ42/40, NfL and GFAP). CSF MTBR‐tau243 has a higher correlation with Tau PET SUVR (r = 0.74, WCGROI) vs. amyloid PET Centiloids (r = 0.39, WCG ROI). Levels of CSF MTBR‐tau243 increased over time in early AD (placebo). Treatment with lecanemab slowed the rate of increase of MTBR‐tau243 vs. placebo, with a greater separation from placebo observed at 18 month (77 weeks) vs. 12 months (53 weeks). While the results have limitations due to sample size and do not reach statistical significance, at 18 months lecanemab slowed the rate of increase in CSF MTBR‐tau243 by 44% vs. placebo.

**Conclusion:**

These results support that CSF MTBR‐tau243 is a specific biomarker of tau tangle pathology and are consistent with the effects of lecanemab on Tau PET, which similarly showed a slowing of tau accumulation vs. placebo. These results demonstrate that lecanemab‐associated amyloid removal slows the formation of tau pathology in the brain.

